# Genome-Wide Identification and Expression Analysis of Cutinase Gene Family in *Rhizoctonia cerealis* and Functional Study of an Active Cutinase RcCUT1 in the Fungal–Wheat Interaction

**DOI:** 10.3389/fmicb.2018.01813

**Published:** 2018-08-07

**Authors:** Lin Lu, Wei Rong, Sebastien Massart, Zengyan Zhang

**Affiliations:** ^1^National Key Facility for Crop Gene Resources and Genetic Improvement, Institute of Crop Sciences, Chinese Academy of Agricultural Sciences, Beijing, China; ^2^Laboratory of Integrated and Urban Phytopathology, Gembloux Agro-Bio Tech–University of Liège, Gembloux, Belgium

**Keywords:** cutinase, compatible interaction, *Rhizoctonia cerealis*, virulence factor, wheat (*Triticum aestivum L*.)

## Abstract

Wheat (*Triticum aestivum* L.) is a staple food of more than 50% of global population. *Rhizoctonia cerealis* is the causal agent of sharp eyespot, a devastating disease of cereal crops including wheat. Cutinases produced by fungal pathogens play important roles in host-pathogen compatible interactions, but little is known about cutinases in *R. cerealis*. In this study, we identified a total of six cutinase encoding genes from *R. cerealis* genome, designated as *RcCUT1–RcCUT6*, analyzed their expression patterns during the infection, and determined virulence role for RcCUT1. All the proteins, RcCUT1–RcCUT6, contain a highly conserved GYSKG motif and another conserved C-x(3)-D-x(2)-C-x(2)-[GS]-[GSD]-x(4)-[AP]-H motif in the carbohydrate esterase 5 domain. The RcCUT1, RcCUT2, RcCUT4, and RcCUT5 are predicted to be secreted proteins containing four cysteine residues. These six cutinase genes had different expression patterns during the fungal infection process to wheat, among which *RcCUT1* was highly expressed across all the infection time points but *RcCUT6* was not expressed at all and the others were expressed only at certain time points. Further, RcCUT1 was heterologously expressed in *Escherichia coli* to obtain a purified protein. The purified RcCUT1 was shown to possess the cutinase activity and be able to induce necrosis, H_2_O_2_ accumulation, and expression of defense-related genes when infiltrated into wheat and *Nicotiana benthamiana* leaves. In contrast, RcCUT1 protein with serine mutation at the first motif had no cutinase activity, consequently lost the ability to induce necrosis. Noticeably, application of the purified RcCUT1 with *R. cerealis* led to significantly higher levels of the disease in wheat leaves than application of the fungus alone. These results strongly suggest that RcCUT1 serves as a virulence factor for the fungus. This is the first investigation of the cutinase genes in *R. cerealis* and the findings provide an important insight into pathogenesis mechanisms of *R. cerealis* on wheat.

## Introduction

Plants have evolved two layers of innate immune systems to defend against microbial attacks, including pathogen-associated molecular pattern (PAMP)-triggered immunity (PTI) and effector-triggered immunity (ETI) (Ausubel, 2005; Chisholm et al., 2006; Jones and Dangl, 2006). PTI is the first layer of defense and launches when plant cell recognizes PAMP through its membrane-localized pattern-recognition receptors. PTI is a relatively weak but broad-spectrum immune response against microbial pathogens. Fungal early infection on plant cell surface releases small molecules, such as cutin monomer, known as damage association molecular pattern (DAMP), which can also trigger PTI-like response (Schweizer et al., 1996a,b; Parker and Köller, 1998; Chassot and Metraux, 2005; Park et al., 2008; Gui et al., 2018). Successful pathogens utilize arsenals of secreted effectors to overcome and interfere with PTI rendering plant susceptible. However, some plants carry intracellular immune receptors which can directly or indirectly recognize pathogen effectors to trigger the second layer of defense, known as ETI. ETI is often observed as a hypersensitive response (HR), a programmed plant cell death at the infection site, which is believed to arrest the fungal growth (Jones and Dangl, 2006). Both PTI and ETI involve plant cell wall reinforcement, biosynthesis of antimicrobial compounds, generation of reactive oxygen species (ROS), and expression of defense genes (Dixon et al., 1994; Ebel and Mithofer, 1998; Garcia-Brugger et al., 2006).

The plant cuticle is mainly composed of cutin, a mixture of hydroxy fatty acid polymers with waxes. It constitutes the first physical defensive barrier against environmental stresses and pathogen invasion (Chen S. et al., 2013). However, most plant pathogens produce cutinases that can hydrolyze cutin facilitating the fungal penetration into their hosts (Maddock, 1979; Koller et al., 1991; Kolattukudy et al., 1995). Cutinases are serine esterases belonging to the α/β hydrolase superfamily, and they possess two conserved motifs of GYSQG and C-x(3)-D-x(2)-C-x(2)-[GS]-[GSD]-x(4)-[AP]-H and a classical Ser-His-Asp catalytic triad (Skamnioti et al., 2008; Chen S. et al., 2013). The fungal cutinases may be involved in fungal spore attachment to plant surfaces, appressorium differentiation, and/or carbon acquisition during saprophytic growth (Deising et al., 1992; Stahl and Schafer, 1992; Francis et al., 1996; Gilbert et al., 1996; Morid et al., 2009; Koschorreck et al., 2010; Zhang et al., 2014). In some phytopathogen species, such as *Fusarium solani, Colletotrichum gloeosporioides*, and *Pyrenopeziza brassicae*, cutinases have been shown to be important for host–pathogen compatible interaction because mutation of some cutinase genes can reduce virulence or eliminate pathogenicity on host plants (Rogers et al., 1994; Li et al., 2003; Liu et al., 2016; Wang et al., 2017). Most fungi have multiple copies of cutinase genes and the role of each cutinase gene in disease could be different (Sweigard et al., 1992; Skamnioti and Gurr, 2007; Liu et al., 2016). For instance, in *Magnaporthe grisea*, disruption of the *cut1* gene did not affect the fungal pathogenicity (Sweigard et al., 1992), while the disruption of the *cut2* gene decreased appressorium differentiation and host penetration, thus affecting fungal virulence (Skamnioti and Gurr, 2007).

Wheat (*Triticum aestivum* L.) production is essential for global food security since it is a staple food of more than 50% of world’s population (Moore et al., 2015; Kiran et al., 2016). The necrotrophic fungus *Rhizoctonia cerealis* van der Hoeven (*R. cerealis*) is the causal agent of sharp eyespot in wheat, a disease mainly on the stem base of wheat plants. Sharp eyespot can affect both the quality and yield (∼10–40%) of wheat (Hamada et al., 2011; Lemańczyk and Kwaśna, 2013). The disease has a global distribution, including Asia, Oceania, Europe, North America, and Africa (Pitt, 1966; Chen L. et al., 2008; Hamada et al., 2011; Chen J. et al., 2013; Lemańczyk and Kwaśna, 2013; Ji et al., 2017). Since the late 1990s, epidemics of wheat sharp eyespot have become very common in China, where more than 6.67 million hectares of wheat plants can be infected by *R. cerealis* annually (Chen L. et al., 2008; Chen J. et al., 2013; Zhu et al., 2015). Sharp eyespot caused by the same fungus can also occur on other cereal crops such as barley, oats, and rye (Van Der Hoeven and Bollen, 1980; Lemańczyk and Kwaśna, 2013). In addition, the fungus can also infect other important economical crops and bioenergy plants, causing root rot disease of sugar beet, cotton, potato, and several legumes, and yellow patch of turfgrasses (Burpee et al., 1980; Tomaso-Peterson and Trevathan, 2007).

*Rhizoctonia cerealis* belongs to the binucleate *Rhizoctonia* subgroup AG-D I (Li et al., 2014). Previous researches on *R. cerealis* mainly focused on the disease geographical distribution, pathogen identification, life cycle, disease symptoms, fungal classification, and population structure of populations (Sharon et al., 2006; Hamada et al., 2011; Li et al., 2014, 2017; Ji et al., 2017). Although *R. cerealis* is a devastating fungal pathogen, its pathogenesis remains largely unknown, which might be due to the lacking of the whole genomic sequence and the effective methods for the stable fungal transformation. Recently, we have completed the genome sequencing of the *R. cerealis* strain Rc207 and finished genome *de novo* assembling and annotation (Zhang et al., unpublished data). In this study, we characterized the cutinase genes in the assembled *R. cerealis* genome, examined their expression patterns, and investigated the function of the most important one, designated as RcCUT1, in the fungal pathogenesis. The results reveal that RcCUT1 is an important virulence factor for *R. cerealis*.

## Materials and Methods

### Fungal Strains, Plant Materials, and Growth Conditions

The fungus *R. cerealis* strain Rc207, kindly provided by Professor Jinfeng Yu at Shandong Agricultural University, was a highly aggressive strain collected in the Northern-China (Ji et al., 2017). The strain was maintained on potato dextrose agar (PDA) at 4°C. To conduct pathogenicity test, the mycelia plug was made and inoculated on new PDA plates or potato dextrose liquid culture, which were then cultivated at 25°C for 10 days before the inoculation.

Wheat line Wenmai6 was susceptible to *R. cerealis* infection. Wheat plants were grown in 13 h light (∼22°C)/11 h dark (∼10°C) regime. At their tillering stage, the second base sheath of each wheat plant was inoculated with small toothpick fragments harboring well-developed mycelia of *R. cerealis* (Chen L. et al., 2008).

*Nicotiana benthamiana* plants were grown under standard glasshouse conditions at 25°C with a cycle of 12 h light and 12 h dark.

### Identification of Cutinase Genes in *Rhizoctonia cerealis*

The members of candidate cutinase gene family were identified using BlastP with an *E* < 1*e*^-10^ from *R. cerealis* Rc207 genome sequence (unpublished data). The codes indicating the enzyme classes were those defined by the CAZyme database^1^. Secreted proteins were identified using three programs that are commonly used to identify protein localization as previously described (James et al., 2014). Subcellular localization, secretion status, and transmembrane domains were predicted with Phobius^2^, SignalP v4.1^3^, and WolfPSort v0.2^4^. Putative extracellular proteins containing signal peptide and no transmembrane domains were identified as secreted proteins. Multiple alignments were made with the Clustal W2 program^5^. Amino acid sequences used for conserved residues analysis were as indicated (**Supplementary File S1**). A neighbor-joining (NJ) tree was constructed using MEGA (version 7.0) program.

### DNA and RNA Extraction and cDNA Synthesis

Total DNA of the fungus was extracted from the mycelia of *R. cerealis* 207 and wheat genomic DNA was extracted with a modified cetyl-trimethyl-ammonium bromide extraction method (Nicholson and Parry, 1996). Total RNAs from the *R. cerealis* Rc207 and the pathogen-inoculated stems or RcCUT1-treated leaves of wheat plants were extracted using the TRIzol (Invitrogen, United States) according to the manufacturer’s instruction. They were subjected to digestion with RNase-free DNase I (Takara, Japan) and purification (Zhang et al., 2007). Reverse transcription was carried out according to the RNA PCR Kit 3.0 instructions (Takara, Japan). Five micrograms of purified RNA from each sample was used as the template for synthesizing the first-strand cDNA by using the SuperScript II First-Strand Synthesis Kit (Invitrogen, United States).

### Real-Time Quantitative PCR (RT-qPCR) Analysis

To investigate the expression patterns of the cutinase genes during the infection process in wheat, *R. cerealis* Rc207 RNAs were extracted from the pathogen inoculated stems of wheat at five different infection time points (18, 36, 72, 96, and 240 h post-inoculation, hpi) with *R. cerealis* Rc207 and from *R. cerealis* Rc207 *in vitro*. The specific RT-qPCR primers for each cutinase gene were designed based on the specific sequences using homologous sequence alignment to distinguish each other. All the primers in the study are listed in **Supplementary Table S1**.

The RT-qPCR was performed using an ABI 7500 RT-PCR system (Applied Biosystems, United States) following the procedure described in Dong et al. (2010). The relative expression of the target genes in *R. cerealis* or wheat was calculated using the 2^-ΔΔ*C*_T_^ method (Livak and Schmittgen, 2001), where the wheat *Actin* gene (*TaActin*) or the *R. cerealis Actin* gene (*RcActin*) was used as the internal reference. Three independent biological replications were performed for each RNA sample/primer combination.

### Heterologous Expression of RcCUT1

The full coding sequence of the *RcCUT1* gene was sub-cloned into the *Bam*HI site of pMAL-c5x vector and fused with the maltose-binding protein (MBP) tag in the vector (New England BioLabs, United Kingdom), which resulted in the expression vector pMBP-RcCUT1. For site-directed mutant of RcCUT1, the Ser-110, critical for the enzymatic activity of RcCUT1, was replaced by alanine. The positive clones with the *MBP-RcCUT1* recombinant gene and the mutant were identified by means of the gene-specific PCR and confirmed by sequencing of the gene. The resulting pMBP-RcCUT1 and mutant pMBP-RcCUT1-S110A fusion constructs were transformed into competent cells of *Escherichia coli* (*E. coli*) *BL21* (DE3), respectively. The recombinant MBP-RcCUT1 and MBP-RcCUT1-S110A proteins were separately expressed after treatment with 0.05 mM isopropyl-β-D-thiogalactopyranose at 16°C for 19 h, and purified using Amylose resin (New England BioLabs, United Kingdom). Protein purity and molecular weight were determined by using sodium dodecyl sulfate-polyacrylamide gel electrophoresis (SDS–PAGE).

### Cutinase Enzyme Activity Assays

Cutinases are defined as extracellular serine esterases that break the ester bond of cutin, a heteropolymer of esters of hydroxylated fatty acids, derived from the cuticle of plant (Parker and Köller, 1998). Cutinase activity in MBP-RcCUT1 can be quantified in an indirect way by using the method previously described (Chen S. et al., 2008). One unit of enzyme activity is defined as the production of 1 μM of *p*-nitrophenol per minute. The standard assay was measured at 20°C in a final volume of 1 mL containing 1 mM *p*-nitrophenyl butyrate (*p*NPB), the enzyme, and the assay buffer (20 mM Tris-HCl, 10 mM NaCl, and 50 mM sodium taurodeoxycholate, pH 8.0). The reaction was initiated by the addition of *p*NPB. The hydrolysis of *p*NPB was spectrophotometrically monitored for the formation of *p*-nitrophenol at 405 nM.

### Cell Death-Inducing Activity of RcCUT1 Protein

Cell death-inducing activity of the heterologously expressed protein was assayed by infiltrating samples (25 μL) into the detached leaves of 2-months-old wheat plants (Zheng et al., 2013; Ma et al., 2015). To determine the minimum concentration required, 25 μL of a serially diluted protein solution (2.5, 5, or 10 μM) was infiltrated into wheat leaves. MBP-tag protein was used as a negative control.

### Diaminobenzidine (DAB) Staining for Detection of H_2_O_2_

Hydrogen peroxide (H_2_O_2_) in plants was detected by DAB staining as previously described method (Thordal-Christensen et al., 1997). *N. benthamiana* leaves treated by MBP-RcCUT1 were sampled, then immediately vacuum-infiltrated with solution of 1 mg ml^-1^ DAB-HCl (pH 3.8) at 25°C. They were placed under dark for 8 h, subsequently were boiled for 5 min in 95% ethanol (Lee et al., 2002).

### Application of the Purified RcCUT1 in Disease Assay

In a detached-leaf inoculation assay, fully expanded secondary leaves (at the tillering stage of the 2-months-old wheat) were injected with MBP-RcCUT1 (25 μL). After the protein was completely infiltrated for 6 h, the leaves were inoculated with 50 μL mycelium suspension of Rc207 nearly to the MBP-RcCUT1 infiltrated. The leaves were then placed in Petri dishes containing filter paper saturated with sterile distilled water and kept under a 16 h day/8 h night regime at 25°C. Pictures of the lesions were taken at 3 days post-inoculation (dpi) with Rc207, and lesion areas were measured by length × width. Each experiment was performed with six leaves and repeated three times. RT-qPCR was used to quantify the ratio of host-to-pathogen RNA sequences, employing primers specific for *R. cerealis* (*RcActin*) (**Supplementary Table S1**). Three independent biological replicates were conducted.

## Results

### Identification of the Cutinase Genes in the *R. cerealis* Genome

**FIGURE 1:**
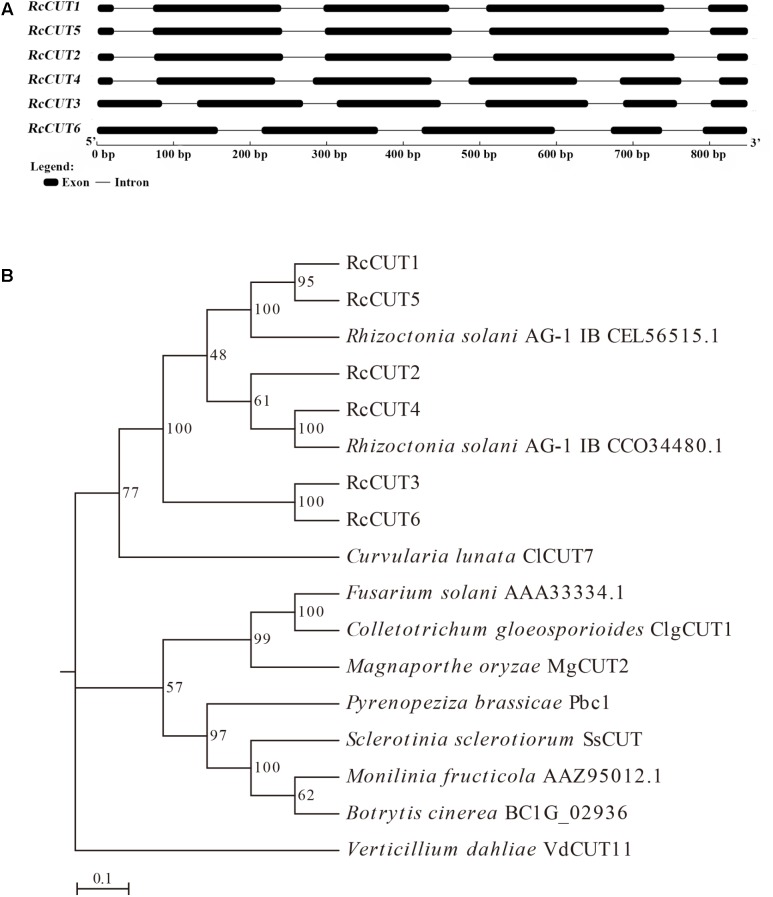
Structures of *RcCUT* genes in *Rhizoctonia cerealis* and their phylogenetic relationships. **(A)** Exons and introns are indicated by black boxes and lines, respectively. The 5′–3′ scale indicates the DNA sequence size. The names of the *RcCUT* genes and intron-exon structures are indicated at the left and right sides, respectively. **(B)** Phylogenetic relationships between cutinases from *R. cerealis* and other fungi. The phylogeny was constructed by Mega 7.0 using neighbor-joining method (parameters: 1,000 bootstraps).

BLASTP searches of the CAZyme database of the *R. cerealis* Rc207 genome identified a total of six cutinase domain-containing proteins, which were designated as RcCUT1–RcCUT6. The protein sequence analysis indicated that they all contain the carbohydrate esterase 5 (CE5) domain (**Table 1**). Using gene specific primers (**Supplementary Table S1**), we cloned and verified the full lengths of coding sequence for all six cutinase genes. The cutinase genes have four to five introns with the length of ORFs ranging from 609 to 738 bp (**Figure 1A** and **Table 1**). Among them, RcCUT6 has the smallest size (21.1 kDa) consisting of 203 amino acid (AA) residues and RcCUT3 is the largest composed by 246 AA residues (**Table 1**). Their isoelectric points (PI) ranged from 5.53 to 8.95 (**Table 1**). Based on signal peptide predictions, four cutinases, including RcCUT1, RcCUT2, RcCUT4, and RcCUT5, were predicted to be a secreted protein. All cutinase proteins except RcCUT6 contained four cysteines (**Table 1**).

**Table 1:** Characteristics of six cutinase genes (*RcCUTs*) in *Rhizoctonia cerealis.*

Gene name	ORF length (bp)	Amino acid (aa)	Molecular weight (kD)	Isoelectric point (pI)	No.Cys	Secreted	CAZYmes
*RcCUT1*	636	212	21.5	8.95	4	Yes	CE5
*RcCUT2*	627	209	21.8	8.43	4	Yes	CE5
*RcCUT3*	738	246	25.9	8.64	4	No	CE5
*RcCUT4*	624	208	21.4	8.48	4	Yes	CE5
*RcCUT5*	633	211	21.6	7.76	4	Yes	CE5
*RcCUT6*	609	203	21.1	5.53	3	No	CE5

Protein sequence alignment was conducted among all the six *R. cerealis* cutinases, and additional 11 cutinase proteins from other plant pathogens retrieved from GenBank (**Supplementary File S1** and **Supplementary Figure S1**). All the six *R. cerealis* cutinases have two conserved motifs that are important for the enzyme activity of cutinases, including C-P-x-[QTA]-x-[FIL]-[VAS]-x-[GS]-G-Y-S-K-G motif (enzymatic activity site, S = serine) and C-x(3)-D-x(2)-C-x(2)-[GS]-[GSD]-x(4)-[AP]-H motif (enzymatic activity sites, D = aspartic acid and H = histidine). These motifs are similar to the cutinase proteins from other phytopathogens (**Supplementary Figure S1**). In addition, we examined all 53 cutinase protein sequences from the *Rhizoctonia solanis* (*R. solani*) strains AG1-IB, AG2-IIIB, AG3, AG8, and 123E that all have been sequenced, and found that they all possess the GYSKG motif and less conserved C-x(3)-D-x(2)-C-x(2)-[GS]-[GSD]-x(4)-[AP]-H motif (**Supplementary File S2** and **Supplementary Figure S2**). To understand the evolutionary relationship between RcCUTs and cutinases from other plant fungal pathogens, a phylogenetic tree was constructed using the neighbor-joining phylogeny (Saitou and Nei, 1987). The phylogenetic tree results showed that the six *R. cerealis* cutinases, *R. solani* cutinases, and *Curvularia lunata* ClCUT7 were clustered into the same group (**Figure 1B**).

### Expression Patterns of *RcCUT1–RcCUT6* During the Infection

**FIGURE 2:**
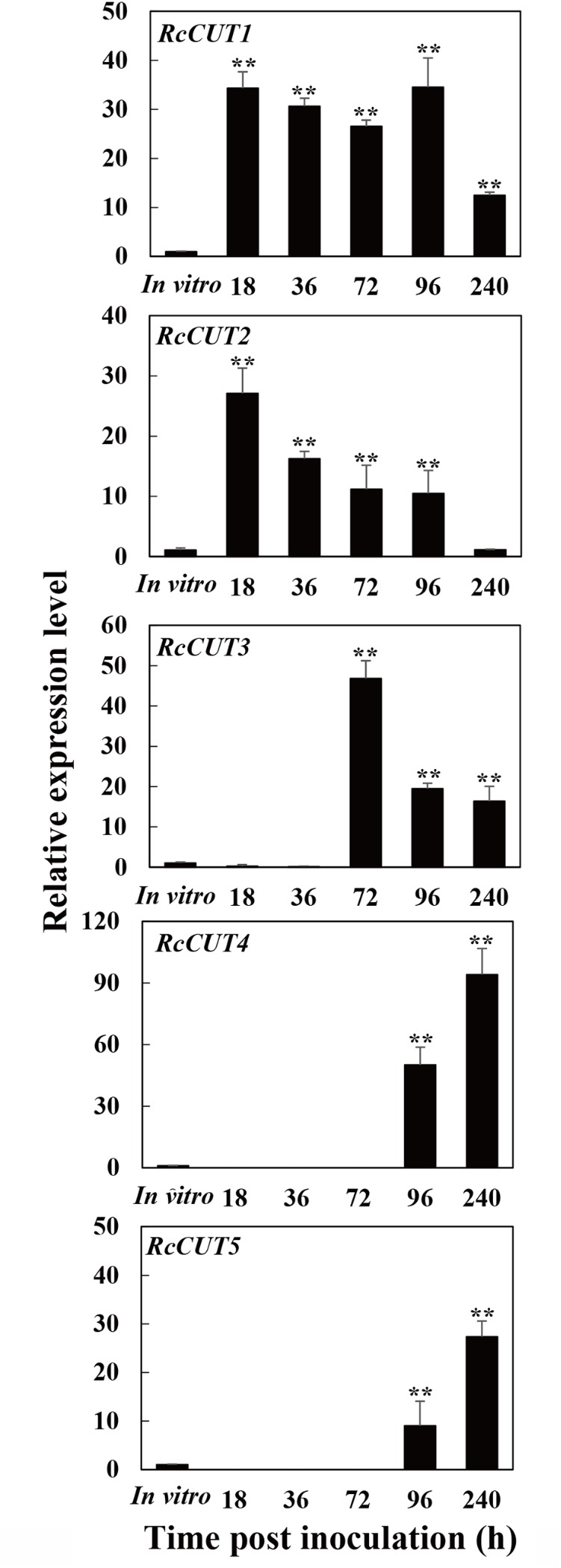
Expression patterns of the *RcCUT* genes in *Rhizoctonia cerealis* during the infection to wheat stems. The *R. cerealis Actin* gene was used as an internal control to normalize the data. The expression of each cutinase gene was investigated during the infection process to wheat stems, including five different infection time points (18, 36, 72, 96, and 240 hpi) and compared to that *in vitro* culturing. Error bars were calculated based on three replicates. Asterisk ^∗∗^ indicates significant difference between the pathogen-inoculated sample and *in vitro* sample (*t*-test; *P* < 0.01).

The expression of each cutinase gene was investigated during the infection process to wheat stems, including five different infection time points (18, 36, 72, 96, and 240 hpi) and compared to that *in vitro* culturing. All the six cutinase genes, *RcCUT1–RcCUT6*, had very low level of expression *in vitro* culture; In contrast, except *RcCUT6*, *RcCUT1–RcCUT5* had significant higher expressional levels *in planta* during the infection at all or some time points when compared to the *in vitro* samples (**Figure 2**). In addition, the five cutinase genes had distinct expression patterns during the infection. Most noticeably, *RcCUT1* had a very high transcriptional level for all the tested infection time points. The transcriptional level of *RcCUT2* was also relatively higher for most of infection time points, but it decreased as time progressed. In comparison, *RcCUT3*, *RcCUT4*, and *RcCUT5* had relatively high transcriptional levels during late time points (**Figure 2**). Lastly, *RcCUT6* had no detectable expression at all five tested infection time points. Because *RcCUT1* is highly expressed among all the tested time points, we suspected that it might be importantly involved in host–pathogen interaction for the disease, and thus we further investigate its function.

### Heterologous Expression and Cutinase Activity of the RcCUT1 Protein

**FIGURE 3:**
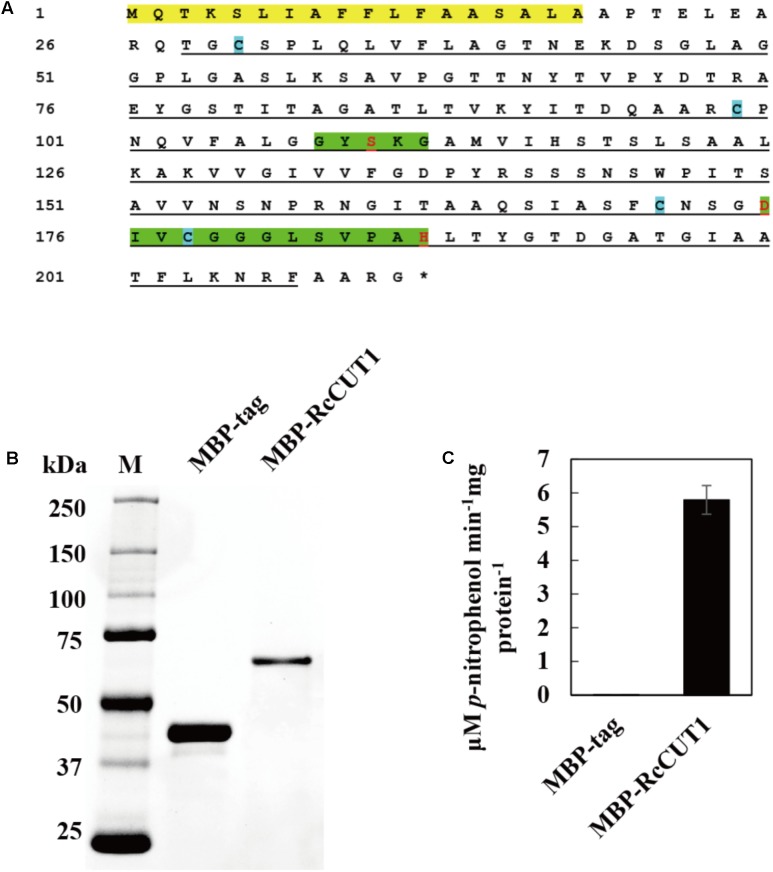
Heterologous expression and cutinase activity of RcCUT1 from *Rhizoctonia cerealis*. **(A)** The deduced amino acid sequence of RcCUT1. Yellow part represents signal protein domain, green space indicates the two highly conserved domains of cutinase, the cutinase domain is marked by black lines, and blue cube represents the cysteine. **(B)** Sodium dodecylsulphate-polyacrylamide gel electrophoresis (SDS-PAGE) of the purified enzymes of RcCUT1 from *R. cerealis*. **(C)** Enzyme activity of RcCUT1 was measured at 20°C using *p*-NPB as substrate. Standard errors are shown.

The predicted RcCUT1 protein consists of a putative signal peptide at 1–18 AA residues, the 28–207 AA residue region possibly containing the cutinase domain, the conserved GYSKG motif at the 108–112 region, and another motif at amino acids of 175–187 (**Figure 3A**). The RcCUT1 shared 68.40% protein sequence identity with a cutinase in *R. solani* (GenBank Accession Number CEL56515.1, function not confirmed yet), 30.49% identity to SsCUT, an elicitor from *Sclerotinia sclerotiorum* (Zhang et al., 2014), 26.27% identity to ClCUT7 (a function-known cutinase in *C. lunata* involved in pathogenicity, Liu et al., 2016), and 17.28% identity to VdCUT11 (Gui et al., 2018).

To obtain purified RcCUT1 protein, the MBP-RcCUT1 recombinant protein was expressed in *E. coli*. After purification, the recombinant MBP-RcCUT1 protein was examined using SDS–PAGE. The purified recombinant protein MBP-RcCUT1 migrated as a single band with an estimated molecular mass of 63 kDa on SDS-PAGE (**Figure 3B**). The cutinase activity assays showed that the purified MBP-RcCUT1 had the enzymatic activity for hydrolyzing *p*NPB substrate while MBP-tag had no activity (**Figure 3C**). This suggests that RcCUT1 is a functional cutinase.

### RcCUT1 Induces Cell Death in the Treated Leaves of Wheat and *N. benthamiana*

**FIGURE 4:**
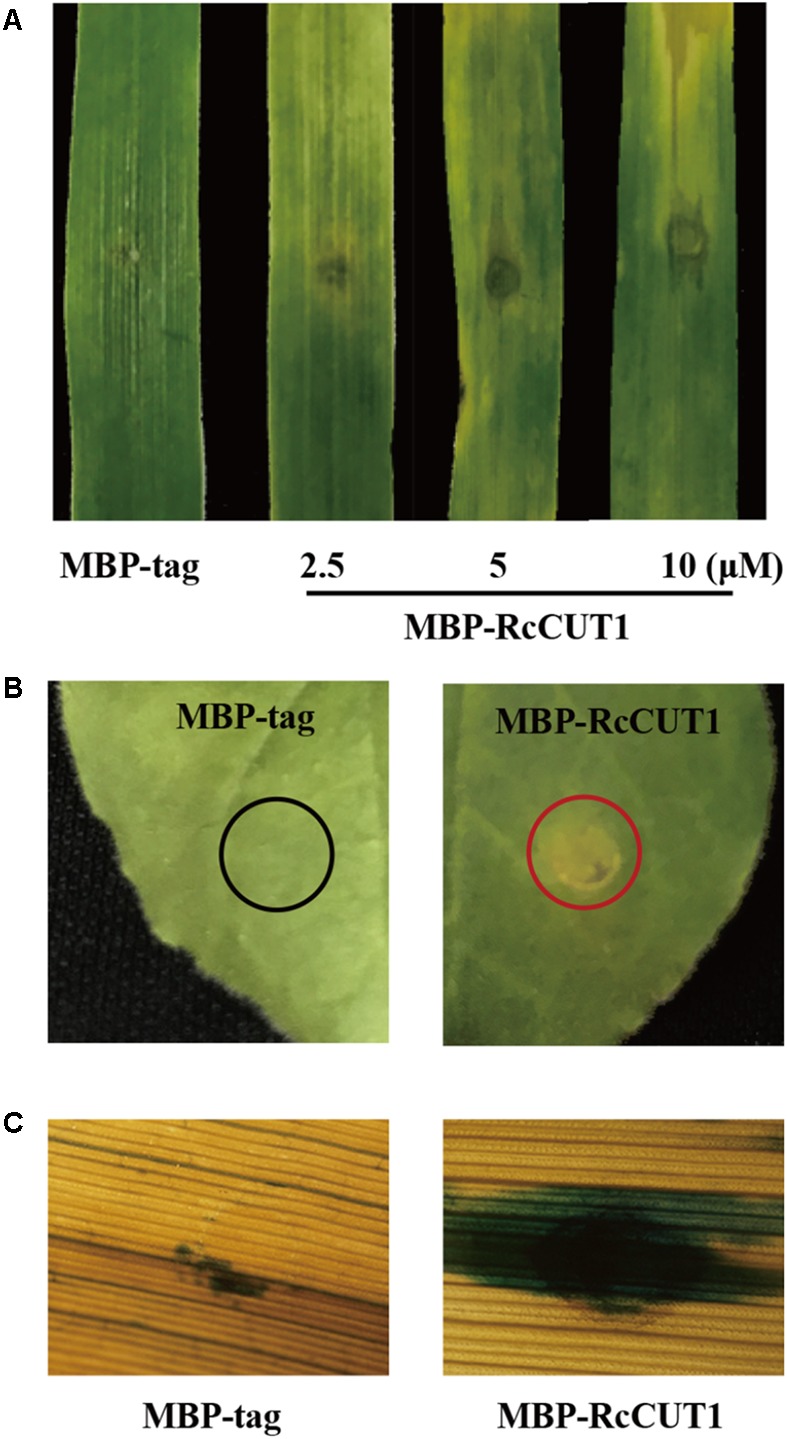
The cell death induced by RcCUT1 in wheat and *N. benthamiana* leaves. **(A)** Wheat leaves 3 days post-inoculation with the MBP-RcCUT1 (2.5, 5, and 10 μM) and the MBP-tag solution (5 μM) as control. **(B)** *N. benthamiana* leaves with MBP-RcCUT1 treatment (5 μM). The red and black circles indicate cell death and no cell death, respectively. **(C)** Wheat leaves with MBP-RcCUT1 treatment (5 μM) stained by trypan blue. Dead wheat leaf cells were stained by trypan blue.

Many fungal cutinases have been shown to have the ability to induce programmed cell death in plant (Zhang et al., 2014; Gui et al., 2018). The purified MBP-RcCUT1 protein was infiltrated into wheat leaves at the concentrations of 2.5, 5, or 10 μM to test its ability to induce cell death. As shown in **Figure 4A**, necrosis and/or chlorosis was clearly observed in the MBP-RcCUT1 infiltrated area on the wheat leaves at 3 days after infiltration with all three concentrations, whereas MBP-tag infiltration did not cause any visible necrotic or chlorotic symptoms. We further tested the induced cell death-inducing activity of RcCUT1 in *N. benthamiana*, and the similar results were observed with the MBP-RcCUT1, being able to induce necrosis/chlorosis, but not with MBP-tag (**Figure 4B**). The necrotic symptom induced by MBP-RcCUT1 in *N. benthamiana* leaves was similar to the necrotic symptom induced by VdCUT11 of *Verticillium dahliae* (Gui et al., 2018). In addition, trypan blue staining showed the occurrence of plant cell death in the necrotic areas induced by MBP-RcCUT1 on wheat leaves (**Figure 4C**).

### The Cell Death-Inducing Activity of RcCUT1 Is Dependent on Cutinase Activity

**FIGURE 5:**
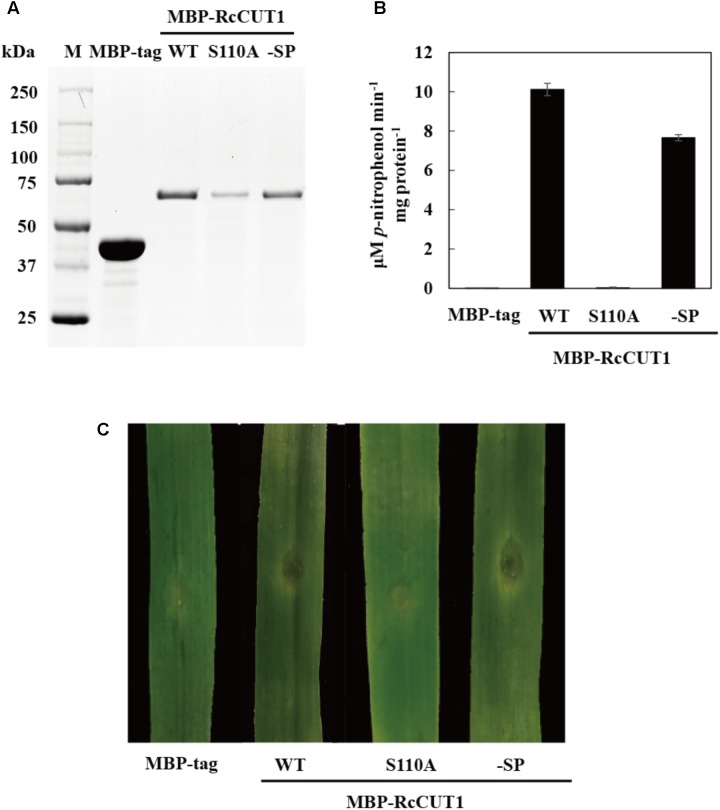
The enzymatic activity is required for the cell death-inducing activity of RcCUT1. **(A)** Sodium dodecylsulphate-polyacrylamide gel electrophoresis (SDS-PAGE) of the purified enzymes of wild-type MBP-RcCUT1 and its mutants. MBP-RcCUT1 S110A, site-directed mutant; MBP-RcCUT1-SP, signal peptide deletion mutant; MBP-tag, as the check sample. **(B)** Enzyme activity of MBP-RcCUT1 variants were measured at 20°C using *p*-NPB as substrate. Standard errors are shown. **(C)** Detection of the cell death-inducing activities of MBP-RcCUT1 variants in wheat leaves.

The three conserved AA residues (Ser, Asp, and His) in cutinase enzymatic domain are essential for the function to catalyze the degradation of the plant cuticle (Chen S. et al., 2013). These three catalytic residues were highly conserved in RcCUT1, including Ser-110 in the first conserved motif, Asp-175 and His-187 in the second motif (**Figure 3A**). To assess the relationship between the cutinase activity and cell death-inducing activity of RcCUT1, the conserved Ser-110 was changed to alanine (Ala, A). Compared to the wild type (WT), the RcCUT1-S110A mutant protein had lost nearly all cutinase activity based on *p*NPB substrate assay (**Figures 5A,B**). Furthermore, the purified mutant RcCUT1-S110A did not result in necrosis development in the infiltrated area on wheat leaves (**Figure 5C**).

We also test if the N-terminal signal peptide is required for necrosis-inducing activity. The predicted signal peptide was deleted from the RcCUT1, which then expressed as the wild type RcCUT1. The results showed that the mutant of signal peptide-deletion (RcCUT1-SP) displayed the same levels of cell death-inducing activity as the wild type RcCUT1 on wheat leaves (**Figure 5C**).

### RcCUT1 Can Induce H_2_O_2_ Production in the Treated Plant Leaves

**FIGURE 6:**
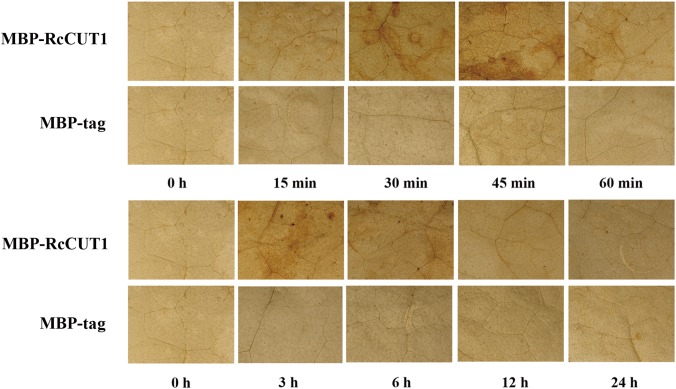
Microscopic observation of H_2_O_2_ accumulation in *N. benthamiana* leaves in response to RcCUT1. H_2_O_2_ accumulation (as indicated by diaminobenzidine staining) appeared in the veins and stomata of MBP-RcCUT1-treated leaves but not in leaves treated with 5 μM MBP-tag solution.

Reactive oxygen species (ROS) accumulation in cells may promote HR and cell death (Temme and Tudzynski, 2009; Ma et al., 2015). H_2_O_2_ is a primary type of ROS and is correlated with the early defense response accompanying cell death (Balbi-Pena et al., 2012; Zhang et al., 2014). Therefore, we examined if treatment of MBP-RcCUT1 protein could induce H_2_O_2_ production in plant leaves. *N. benthamiana* leaves were infiltrated with MBP-RcCUT1 and the control MBP-tag, respectively, and collected at different time points for diaminobenzidine (DAB) staining. H_2_O_2_ accumulation was detected at some time points in *N. benthamiana* leaves infiltrated with MBP-RcCUT1, mainly concentrated in the veins and stomata of *N. benthamiana* leaves (**Figure 6**). However, no obvious (DAB) precipitation was observed for the infiltration with the MBP-tag control (**Figure 6**). We noticed stronger H_2_O_2_ accumulation occurred at 45 min and 3 h after infiltration (**Figure 6**). The two phases of oxidative burst were also observed for *S. sclerotiorum* cutinase treatment in *N. benthamiana* leaves (Zhang et al., 2014).

### RcCUT1 Activates the Expression of Defense-Related Genes in the Treated Wheat

**FIGURE 7:**
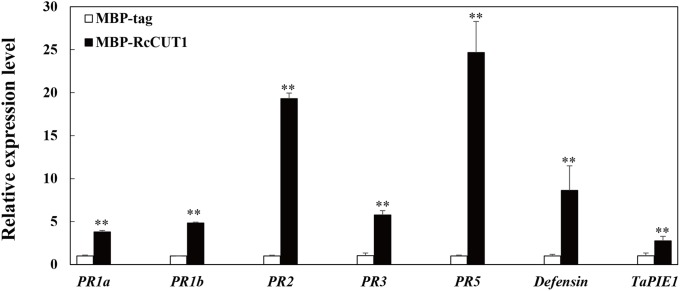
Induction of defense response genes by RcCUT1 from *Rhizoctonia cerealis*. Expression analysis of defense-related genes in wheat leaves 3 days after inoculation with the MBP-RcCUT1 and the check sample MBP-tag at a concentration of 5 μM. The wheat *Actin* gene was used as an internal control to normalize the data. Error bars were calculated based on three replicates. Double asterisk ^∗∗^ indicates significant difference between MBP-RcCUT1 treatment and MBP-tag treatment (*t*-test; *P* < 0.01).

We used RT-qPCR to examine the expression of eight defense-related genes in wheat leaves after treatment with MBP-RcCUT1 or MBP-tag alone. These wheat defense-related genes we investigated included the pathogenesis-related genes *PR1a*, *PR1b*, *PR2*, *PR3*, *PR5*, *defensin*, and an *ethylene-response factor TaPIE1* (Zhang et al., 2012; Liu et al., 2013; Zhu et al., 2014). As expected, the RT-qPCR results showed that all eight genes exhibited significantly elevated expression patterns in wheat leaves infiltrated with RcCUT1 than those infiltrated with MBP-tag (**Figure 7**). Among the eight genes, *PR2* and *PR5* which encode a β-1,3-glucanase and a thaumatin-like protein, respectively, had the much higher transcriptional induction than others after RcCUT1 treatment (**Figure 7**).

### RcCUT1 Contributes to Pathogenicity in *R. cerealis* Infection to Wheat

**FIGURE 8:**
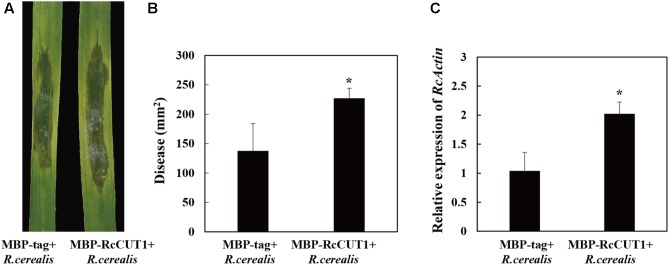
RcCUT1 contributes to pathogenicity in *Rhizoctonia cerealis* infection to wheat. **(A)** MBP-RcCUT1 enhanced pathogenic phenotypes of *R. cerealis* in wheat leaves. Pictures of the lesions were taken at 3 days post-inoculation with the fungus and the lesion area was measured. **(B)** Disease severity measured as area of necrosis induced by *R. cerealis* mycelia on leaves shown in **A**. **(C)** RT-qPCR measurement of the pathogen and wheat RNA ratios was used to determine *R. cerealis* relative biomass in infected plant tissues shown in **A**. Three independent biological replicates were conducted. Error bars were calculated based on three replicates. Asterisk ^∗^ indicates significant difference between MBP-RcCUT1 treatment and MBP-tag treatment (*t*-test; *P* < 0.05).

To shed light on the virulence role of RcCUT1, the liquid mycelia of *R. cerealis* Rc207 were inoculated on the surface of the wheat leaves pretreated by MBP-RcCUT1 or MBP-tag. Disease developments were then recorded as time progressed by measuring the water-soaking area. At 2 dpi, water-soaking lesion was clearly observed on the *R. cerealis* Rc207 inoculated leaves pretreated with the MBP-tag control or MBP-RcCUT1 (**Figure 8A**). However, the disease development was faster and the disease lesions were larger in leaves pretreated with MBP-RcCUT1 than those pretreated with MBP-tag control, and statistical analysis also showed a significant difference between the MBP-RcCUT1 and the control at 3 dpi with *R. cerealis* Rc207 (**Figures 8A,B**). We also measured the fungal relative biomass using RT-qPCR with *R. cerealis Actin* gene in the infected plant tissues at 3 dpi with *R. cerealis* Rc207. As a result, the *R. cerealis* relative biomass in the MBP-RcCUT1-pretreated leaves was significantly higher than in the MBP-tag-pretreated control leaves (**Figure 8C**).

## Discussion

Sharp eyespot, caused by *R. cerealis*, is now considered as one of the most important wheat diseases in China and has also been reported in many other places of the world (Hamada et al., 2011; Chen J. et al., 2013; Lemańczyk and Kwaśna, 2013; Ji et al., 2017). Management of wheat sharp eyespot is nearly impossible because of the lacking of host resistance and poor understanding of host–pathogen interaction in this disease system. The long term goal of our research is to reveal the molecular mechanisms underlying the fungal pathogenicity and infection. To do that, we have sequenced and annotated the whole genome of a highly virulent *R*. *cerealis* strain Rc207, which was collected in China (unpublished data). In this work, we identified a total of six cutinases from the sequenced *R*. *cerealis* genome, characterized their gene structures and expression patterns, and investigated the possible role of cutinases in fungal virulence. *RcCUT1*, one of the cutinase genes, was shown to express at a high level during the whole infection process, possess functional cutinase activity, and able to induce necrosis and defense responses in both wheat and *N. benthamiana*. To our knowledgement, this is the first time to investigate fungal pathogenesis focusing on cutinase genes in *R. cerealis*.

Previous publications have reported 17 cutinase genes in *M. grisea*, 13 in *C. lunata*, 13 in *V. dahlia*, 12 in *Fusarium graminearum*, 11 in *Botrytis cinerea*, four in *Aspergillus nidulans*, and three in *Neurospora crassa* (Skamnioti et al., 2008; Liu et al., 2016; Gui et al., 2018). In *R. solani*, seven cutinase genes in *R. solani* AG8 and only one cutinase gene in *R. solani* AG1 IA were identified, whereas their roles have not been studied yet (Zheng et al., 2013; James et al., 2014). Here, six non-redundant cutinase proteins were identified from *R. cerealis* genome. All the six protein sequences of RcCUT1–RcCUT6 possess the same amino acid sites (GYSKG) in the conserved 1st motif and another conserved C-x(3)-D-x(2)-C-x(2)-[GS]-[GSD]-x(4)-[AP]-H motif. Previous documents reported that in many other pathogenic fungi, cutinase protein sequences contain a highly conserved motif (GYSQG), and a less precise motif (C-x(3)-D-x(2)-C-x(2)-[GS]-[GSD]-x(4)-[AP]-H) that carries aspartate and histidine residues at the active sites (Skamnioti et al., 2008; Chen S. et al., 2013; Liu et al., 2016). Furthermore, we performed the protein sequence alignment among RcCUT1–RcCUT6 and 53 cutinase proteins from five sequenced genomes of *R. solanis* strains and found that these 59 cutinase proteins in *Rhizoctonia* all include the same amino acid sites GYSKG. The GYSKG motif may be a characteristic of the *Rhizoctonia* cutinases (**Supplementary Figure S2**), which is distinct from the GYSQG motif of most cutinases from other fungi, such as *M. grisea*, *F. graminearum*, *B. cinerea, C. lunata*, *and V. dahlia* (Skamnioti et al., 2008; Chen S. et al., 2013; Liu et al., 2016; Gui et al., 2018).

Although *R. cerealis* has six cutinase genes, their expression patterns were quite different. Most remarkably, *RcCUT1* was found to express at very high levels even from the beginning and maintain its high expressional levels for all the tested time points. The transcriptional level of *RcCUT2* was relatively higher for most of infection time points but decreased as infection time progressed. In comparison, transcriptional levels of *RcCUT3*, *RcCUT4*, and *RcCUT5* were high during late infection time points, while *RcCUT6* had no detectable expression at all five infection time points. Early expression has also been observed for some cutinase genes from other fungal pathogens. *ClCUT7*, one of *C. lunata* cutinase genes, was found to express as early as 3 h after inoculation, thus was suggested to be involved in cuticle degradation during the infection (Liu et al., 2016). Similarly, *VdCUT11*, one of cutinase genes from *V. dahlia*, was most significantly up-regulated during the early infection stage (Gui et al., 2018). These data strongly suggest *RcCUT1* may be involved in all stages of fungal infection and colonization. Using the purified RcCUT1 protein, the RcCUT1 protein was clearly demonstrated to possess an *in vitro* cutinase activity to digest cutin polymer. We also showed that RcCUT1 is capable of inducing necrosis (programmed cell death) and triggering defense responses. Most importantly, application of the purified RcCUT1 with *R. cerealis* led to significantly higher level of the disease in wheat leaves than application of the fungus alone. Therefore, it is likely that RcCUT1 functions as a cutinase to degrade wheat cuticle layer helping the fungal penetration in the early stage, and it acts as a fungal effector to induce plant cell death facilitating the late colonization of the necrotrophic fungal pathogen. Additionally, it is interesting to further study roles of RcCUT2, RcCUT4, and RcCUT5 in the compatible interaction between *R. cerealis* and wheat.

Effectors are proteins and small molecules produced by a pathogen to alter the structure and function of host cells (Hogenhout et al., 2009). They are usually secreted and some contain multiple cysteine residues (Saunders et al., 2012). Necrotrophic fungal pathogens utilize an array of effectors to induce plant cell death which may facilitate the growth of the necrotrophic pathogens (Wang et al., 2014). Most cutinases in *R. cerealis*, including RcCUT1, RcCUT2, RcCUT4, and RcCUT5, are predicted to be secreted proteins, each with four cysteine residues, which indicates their potential as a necrotrophic effector. Here, RcCUT1 could induce host cell death, H_2_O_2_ accumulation, the expression of defense-related genes, and contribute to pathogenicity of *R. cerealis.* It is deduced that RcCUT1 may be an effector of the necrotrophic fungus *R. cerealis*. Several cutinases from other fungal pathogens were also shown to have activity as necrotrophic effectors, for example, *VdCUT11* in *V. dahlia* (Gui et al., 2018) and *ClCUT7* from *C. lunata* (Liu et al., 2016). Interestingly, site-directed mutagenesis of serine to alanine in the cutinase domain abolished RcCUT1 cutinase activity, which also eliminated its effector activity. Therefore, the function of RcCUT1 as an effector is dependent on its cutinase activity. This is similar to the report for VdCUT11 (Gui et al., 2018). However, for the *S. sclerotiorum* cutinase SsCUT acting as an elicitor, site-directed mutagenesis of the serine site reduced 99% of cutinase activity but did not affect its ability to trigger plant defense responses (Zhang et al., 2014).

## Conclusion

In conclusion, we identified six cutinase genes from *R. cerealis* genome and revealed their different expression patterns during the infection process. We provided strong evidence that *RcCUT1*, one of cutinase genes in *R. cerealis*, can function as a necrotrophic effector to trigger plant cell death and H_2_O_2_ and contributes to the fungal virulence. These findings facilitate a better understanding of the compatible interaction of the *R. cerealis* and wheat. This study provides a candidate gene for improving wheat varieties with resistance to *R. cerealis* through gene editing technique.

## Author Contributions

ZZ and LL designed the research and wrote the paper. LL and WR performed the genome-identification, cloning, and expression analyses, and serial functional analyses of RcCUT1. SM gave suggestions.

## Disclaimer

This intellectual property in this paper belongs to Institute of Crop Sciences, Chinese Academy of Agricultural Sciences.

## Conflict of Interest Statement

The authors declare that the research was conducted in the absence of any commercial or financial relationships that could be construed as a potential conflict of interest.

## Footnotes

1 http://www.cazy.org/

2 http://phobius.binf.ku.dk/

3 http://www.cbs.dtu.dk/services/SignalP-4.1/

4 http://www.genscript.com/wolf-psort.html

5 http://www.ebi.ac.uk/Tools/msa/clustalw2/

## Supplementary Material

FIGURE S1 Sequence alignment of *Rhizoctonia cerealis* cutinase proteins with 11 known cutinases from other fungi. C-P-x-[QTA]-x-[FIL]-[VAS]-x-[GS]-G-Y-S-[QK]-G and C-x(3)-D-x(2)-C-x(2)-[GS]-[GSD]-x(4)-[AP]-H are marked in black boxes.

FIGURE S2 Alignment of *Rhizoctonia cerealis* cutinase protein sequences with 53 cutinase protein sequences from the *Rhizoctonia solanis* strains AG1-IB, AG2-IIIB, AG3, AG8, and 123E.

TABLE S1 All primers used in this study.

FILE S1 Amino acid sequences of 11 known cutinases from other fungi (*Monilinia fructicola*, *Fusarium solani*, *Curvularia lunata*, *Sclerotinia sclerotiorum*, *Verticillium dahlia*, *Colletotrichum gloeosporioides*, *Botrytis cinereal*, *Pyrenopeziza brassicae*, *Magnaporthe oryzae*, *Rhizoctonia solani* AG-1 IB).

FILE S2 Fifty-three cutinase amino acid sequences from the *Rhizoctonia solanis* strains AG1-IB, AG2-IIIB, AG3, AG8, and 123E.
