# Supplementary figures and images for: Genome-Wide Identification and Expression Analysis of Cutinase Gene Family in Rhizoctonia cerealis and Functional Study of an Active Cutinase RcCUT1 in the Fungal–Wheat Interaction
Source: Front Microbiol. 2018 Aug 7;9:1813. doi: 10.3389/fmicb.2018.01813 (PMC6091245; doi:10.3389/fmicb.2018.01813)

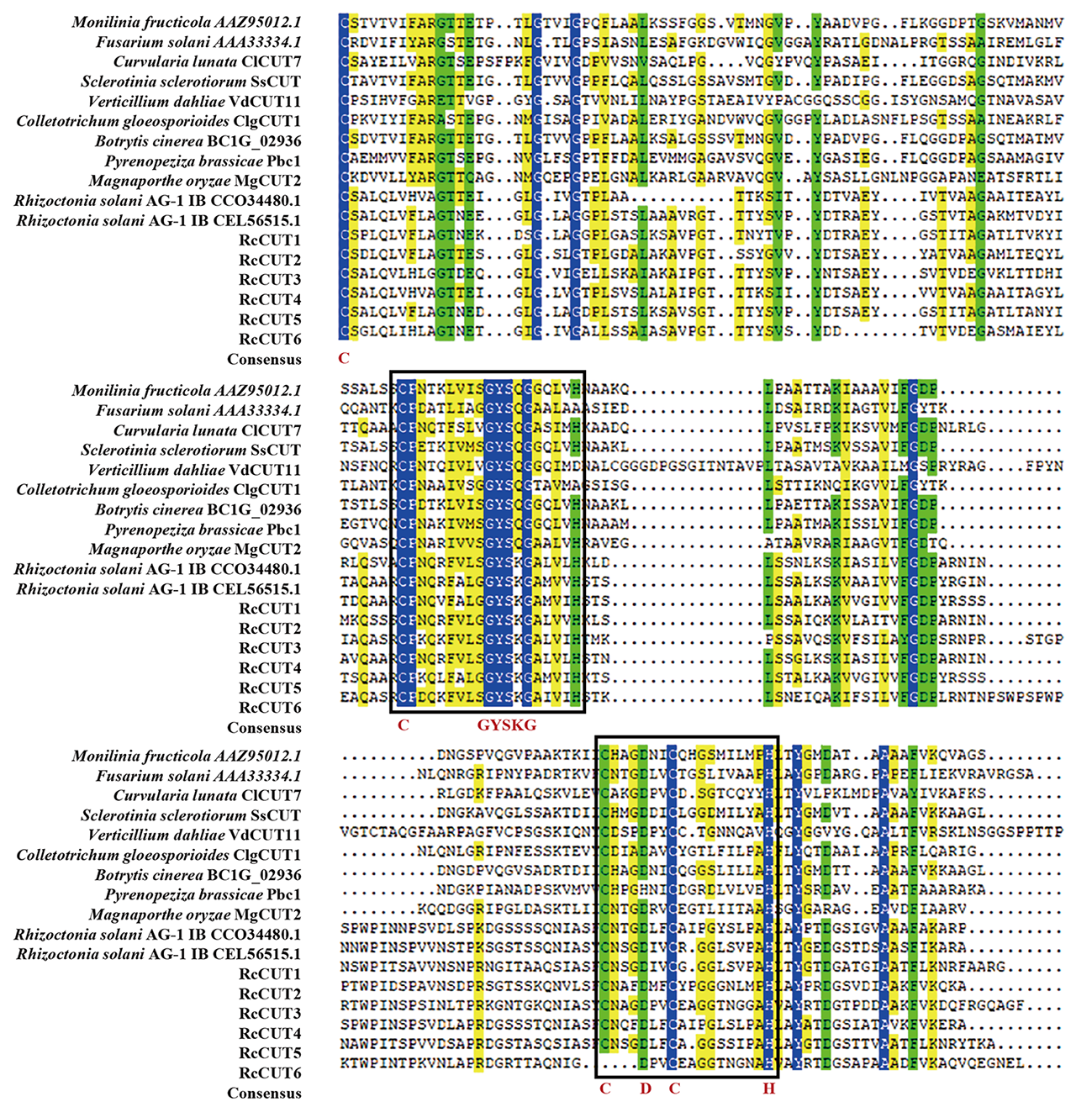

Supplement: FIGURE S1 — Sequence alignment of Rhizoctonia cerealis cutinase proteins with 11 known cutinases from other fungi. C-P-x-[QTA]-x-[FIL]-[VAS]-x-[GS]-G-Y-S-[QK]-G and C-x(3)-D-x(2)-C-x(2)-[GS]-[GSD]-x(4)-[AP]-H are marked in black boxes. [file Image_1.TIF]
